# Fertility desire and associated factors among women of reproductive age living with HIV/AIDS attending antiretroviral therapy clinic in Arba Minch General Hospital, South Ethiopia, 2021

**DOI:** 10.3389/fgwh.2023.1001479

**Published:** 2023-11-08

**Authors:** Disasa Tufa, Biresaw Wassihun, Direslgne Misker, Kassaw Beyene

**Affiliations:** ^1^ Gather Hospital, South Omo Zone, Jinka, Ethiopia; ^2^ Department of Midwifery, College of Health Sciences, Injibara University, Injibara, Ethiopia; ^3^ School of Public Health, College of Medicine and Health Sciences, Arba Minch University, Arba Minch, Ethiopia; ^4^ Department of Midwifery, College of Health Sciences, Arba Minch University, Arba Minch, Ethiopia

**Keywords:** women living with HIV, antiretroviral, fertility desire, Arba Minch, Ethiopia

## Abstract

**Background:**

The fertility desire of women living with HIV to have children can have significant implications for public health. Despite the increase in the number of HIV-positive women, the issues of their fertility desire have not been well-studied. This study aims to assess fertility desire and associated factors among women living with HIV/AIDS.

**Methods:**

A facility-based cross-sectional study was conducted from 1 May to 30 July 2021. The researchers employed a systematic sampling technique. The data were gathered and entered into EpiData 3.1 software, and subsequently exported to the statistical package for social science version 25 for analysis. Binary logistic regression analyses were used to identify the factors involved, and a *p*-value of <0.05 at a 95% confidence level was deemed to be statistically significant.

**Result:**

The findings of this study indicate that 47.8% of women expressed a desire to conceive. Various factors such as parents’ pressure [adjusted odds ratio (AOR) = 4.41, 95% confidence interval (CI): 2.15–9.05], community pressure (AOR = 2.62, 95% CI: 1.30–5.26), being married (AOR = 0.25, 95% CI: 0.09–0.73), having only female offspring (AOR = 2.57, 95% CI: 1.12–5.90), and having HIV seropositive children (AOR = 2.45, 95% CI: 1.23–4.85) had statistically significant association with fertility desire.

**Conclusion:**

The study area exhibited a high level of fertility desire. Various factors can influence fertility desire, including parents’ pressure, community pressure, being married, having only female offspring, and having children who are HIV seropositive. When developing interventions on sexual and reproductive health issues, it is imperative for policymakers and healthcare providers who are working in antiretroviral therapy clinics to take into account the effects of these factors on women living with HIV. When designing and implementing prevention of mother-to-child transmission services, it is important to consider the fertility desires of mothers who are living with HIV.

## Introduction

Fertility desire is a psychological state in which someone has the personal motivation to have a child. Those who have the motivation to have more children in the future have a fertility desire (1).

Human immunodeficiency virus (HIV)/acquired immunodeficiency syndrome (AIDS) remains a major public health problem and affects the fertility desire of people living with HIV/AIDS (PLWHA) worldwide (2). Since the introduction of antiretroviral therapy (ART), the life expectancy of women living with HIV/AIDS (WLWHA) has noticeably increased. However, they are challenged by many reproductive health issues (3). In many areas, the majority of women living with HIV/AIDS who desire additional children have not discussed the issue of fertility desire and childbearing with their healthcare providers (4).

Despite facing financial constraints, lack of support and services, stigma related to HIV, and unsupportive attitudes from healthcare providers, WLWHA remain committed to having children. This is due to their desire to experience motherhood and family life, which outweighs the challenges associated with their HIV status (5, 6).

Hence, many PLWHA desire to have children. In this regard, the guidance provided by healthcare providers plays a critical role in encouraging planned pregnancies, optimizing the prevention of mother-to-child transmission (PMTCT), and reducing fertility-related complications (7).

Previously, policies in many countries discouraged HIV-infected individuals from having children in order to reduce the number of children born with HIV or born to HIV-infected parents, but a more flexible approach toward the reproductive choices of people living with HIV (PLHIV) has now emerged. This shift has been mainly informed by a reproductive rights approach and universal access to PMTCT/ART interventions, as well as the availability of assisted reproductive techniques for HIV-infected people in developed countries, which have dramatically reduced the chances of sexual and perinatal HIV transmission (8, 9).

Studies conducted in Ethiopia have investigated the fertility desires of women living with HIV (WLHIV), revealing a range of 33.4%–56.2% (10, 11). Many infants are infected through mother-to-child transmission (MTCT). Despite the fact that the percentage of pregnant women who receive antiretroviral (ARV) treatment for PMTCT was 92%, there were still 2,700 cases of new HIV infections reported among individuals aged 0–14 years; however, 3,700 cases of new HIV infections in infants were averted (12). Studies revealed that factors such as gender expectations for women to have children and the desires of family members and/or parents and partners for the woman to have a child influence a woman's own desire to conceive. In fact, the perceived desire of one’s partner may play a more crucial role compared with the other factors (7, 13, 14). These multifaceted influences on reproductive decision-making and potential gaps in knowledge highlight the need to explore the processes and the individuals involved in the reproductive decision-making of women. Therefore, this study aims to assess the fertility desire and associated factors among women living with HIV/AIDS.

## Methods and materials

### Study area and design

A facility-based cross-sectional study design was conducted from 1 May to 30 July 2021 in the Arba Minch General Hospital ART clinic in Arba Minch town, the capital city of Gamo Zone. Arba Minch town is located approximately 504 km from Addis Ababa, the capital city of Ethiopia. The town has one governmental zonal hospital, which serves a population of an estimated 118,040 people in the catchment area, as well as one primary hospital and two public health centers. Arba Minch General Hospital started service in 1969. According to the HIMS report of 2021, Arba Minch General Hospital has 266 beds and provides different services such as the Outpatient Department, Inpatient Department, Emergency Services, pharmacy service, radiology service, physiotherapy service, psychiatry service, Maternal Neonatal Child Health service, and ART services by different disciplines. The total number of reproductive age PLHIV following ART in Arba Minch General Hospital is 1,871. Among this group, 931 individuals are women aged 18–49 years.

### Population

#### Source population

The study focuses on reproductive age women who are living with HIV/AIDS and are attending the ART clinic at Arba Minch General Hospital.

#### Study population

The study included women of reproductive age who are living with HIV/AIDS and are attending the ART clinic at Arba Minch General Hospital during the specified study period.

#### Eligibility criteria

The study included all women of reproductive age who were infected with HIV, receiving ART, and attending the ART clinic during the data collection period. The study excluded pregnant women living with HIV/AIDS who experienced severe illness, rendering them unable to provide responses to the questionnaires.

#### Sample size determination

The sample size was determined by using Epi Info version 7.2.3.1 menu STAT CALC, by considering the assumptions of a 95% confidence level, 80% power, and an exposed to unexposed ratio of 1:1. The minimum required sample size is 410. However, to account for a non-response rate of 5%, the adjusted sample size becomes 431.

#### Sampling procedure

The total number of reproductive age WLHIV receiving care at the ART clinic in Arba Minch General Hospital is 1,871. Among those, 931 were women who are aged 18–49. The participants were selected using the systematic random sampling technique. The estimation of the total population of women aged 18–49 living with HIV/AIDS was conducted based on their flow patterns at the ART clinics in the past 6 months. Sampling interval (*K*) was obtained by dividing the total number of women attending the clinic (*n*) by the sample size calculated (931/431 = 2). The first participant was selected randomly, after which subsequent interviews were conducted with every second female participant.

### Study variables

#### Dependent variables

The study focused on the fertility desires of women as the dependent variables.

#### Independent variables

**Sociodemographic characteristics** (age, sex, residence, income, marital/relationship status, education, religion, occupation, ethnicity).

**Children-related factors**: the patient’s current number of living children, biological sex of the children, HIV status of the children, the patient’s knowledge of MTCT, and experiences of child loss due to HIV.

**Health-related factors**: duration of ART, CD4 cell count, viral load, HIV status of the partner, current perceived health condition of women, and contraceptive usage.

**Social-related factors**: partner fertility desire, social support, community pressure, healthcare provider discrimination, and HIV-related stigma.

#### Operational definitions

**Fertility desire**: a psychological state in which someone has the personal motivation to have a child, and those who did not have the motivation to have more children were considered not to have a fertility desire (1).

**HIV-positive women**: all WLWHA who have taken ART care from the study institutions.

**Social support:** the Oslo three-item social support scale, which is considered one of the best predictors of mental health, covering different fields of social support and perceived ways of getting assistance from neighbors. The sum ranges from 3 to 14, and the score indicates varying levels of support, namely, poor support (3–8), moderate support (9–11), and strong support (12–14).

#### Data collection procedures

A semi-structured questionnaire was adapted from the literature that has similar objectives, and a pretest was completed (13). The questionnaire was prepared in the English language, then translated into Amharic, and then retranslated back to English by people who are proficient in both languages to maintain the consistency of the questionnaires. The data were collected by face-to-face interviews using a semi-structured questionnaire that had been pre-tested in Amharic. The questionnaire was administered by two well-trained diploma nurses who are working in the ART clinic, and one BSC nurse was selected for supervision during the data collection period, following a 1-day training session on data gathering techniques. The study assessed the dependent variable of future fertility desire with a binary question: “Would you like to have children in the future?” with response options of “Yes” or “No.”

#### Data quality assurance

A semi-structured data collection tool was used in this study. Data collectors and supervisors were trained on data collection techniques and were briefed on each question included in the data collection tool. A pretest was conducted on 5% (22 questionnaires) of the actual sample size at Chench Hospital, and then the correction was made before the actual data collection. During the data collection procedures, the supervisor and principal investigators reviewed and checked all the collected data on a daily basis for consistency and completeness.

#### Data processing and analysis

The investigators verified the collected data. Subsequently, the data underwent coding, entry, and cleaning processes using EpiData version 3.1 software. Finally, the data were exported into SPSS version 25 for analysis. Next, the information was presented using frequencies, tables, and figures. A bivariate analysis was conducted, and the association between each independent variable and the outcome variable was examined using binary logistic regression, employing a crude odds ratio (COR) with 95% confidence interval (CI). Variables that were significant in the bivariate logistic regression were entered into the multiple regression analysis. The Hosmer–Lemeshow and Omnibus tests were performed to test for model fitness. An adjusted odds ratio (AOR) with 95% CI was estimated to identify the factors associated with fertility desire using multivariable logistic regression analysis. The level of statistical significance was declared at a *p*-value < 0.05.

#### Ethical considerations

Ethical clearance was obtained from the Institutional Research Ethical Board (IRB/2021) of Arba Minch University, College of Medicine and Health Sciences. Permission was secured from the health facility through a formal letter. To keep the confidentiality of the patients’ information, only those personnel who are working in ART units were involved in the data collection. The purpose and processes of the study were explained to all the participants, and written informed consent was obtained from each respondent for permission before interviewing. Confidentiality was maintained and assured by excluding their names from the identification of the study subjects.

## Results

### Sociodemographic characteristics

This study included a total of 427 study participants, with a response rate of 99.1%. The mean (±SD) age of the participants was 31.97 (±7.11) years. More than half (50.6%) of the participants were found within the age group of 30–39 years. In total, 188 (44%) of the study participants were Gamo by ethnicity, 218 (51.1%) were orthodoxy by religion, and 146 (34.2%) were Protestants. In addition, 251 (59%) of the participants were married, while more than one-third (33%) were housewives. A total of 399 (93.4%) of the participants were from the urban area, and 118 (27.6%) of the participants had an elementary education. Regarding incomes, more than half (228, 53.4%) of the participants reported a monthly income of 1,001–5,000 Ethiopian birr (Table 1).

**Table 1:** Sociodemographic characteristics of women living with HIV/AIDS attending ART clinic in Arba Minch General Hospital, South Ethiopia, 2021.

Sociodemographic variables	Had fertility desire	
	Yes (%)	No (%)
Age categories in years (*N* = 427)		
18–29	87 (57.2)	65 (42.8)
30–39	88 (40.7)	128 (59.3)
40–49	29 (49.2)	30 (50.8)
Marital status (*N* = 427)		
Single/unmarried	24 (55.8)	19 (44.2)
Married	149 (59.1)	103 (40.9)
Widowed	16 (24.2)	50 (75.8)
Divorced	15 (22.7)	51 (77.3)
Residence (*N* = 427)		
Rural	11 (39.3)	17 (60.7)
Urban	193 (48.4)	206 (51.6)
Ethnicities (*N* = 427)		
Gamo	102 (54.3)	86 (45.7)
Gofa	38 (55.9)	30 (44.1)
Waliata	19 (37.3)	32 (62.7)
Amhara	21 (39.6)	32 (60.4)
Konzo	5 (23.8)	16 (76.2)
Oromo	10 (45.5)	12 (54.5)
Tigre	5 (38.5)	8 (61.5)
Others, Gurage, and Zayise	4 (36.4)	7 (63.6)
Religion (*N* = 427)		
Orthodox	107 (49.1)	111 (50.9)
Protestant	66 (45.2)	80 (54.8)
Muslim	24 (50)	24 (50)
Catholic	7 (46.7)	8 (53.3)
Educational status (*N* = 427)		
Unable to write and read	12 (36.4)	21 (63.6)
Read and write	34 (42.5)	46 (57.5)
Elementary (1–8 grades)	58 (49.2)	60 (50.8)
Secondary (9–10 grades)	38 (44.2)	48 (55.8)
Preparatory (11–12 grades)	20 (54.1)	17 (45.9
Diploma	30 (56.6)	23 (43.4)
University level	12 (60)	8 (40)
Family income (*N* = 427)		
<500 ETB	4 (40)	6 (60)
500–1,000 ETB	28 (31.5)	61 (68.5)
1,001–5,000 ETB	119 (52.2)	109 (47.8)
≥5,000 ETB	51 (54.8)	42 (45.2)
No income	2 (28.6)	5 (71.4)
Occupational (*n* = 427)		
Student	16 (61.5)	10 (38.5)
Housewife	69 (48.9)	72 (51.1)
Daily laborer	16 (39)	25 (61)
Merchant	25 (53.2)	22 (46.8)
Government Employed	55 (52.9)	49 (47.1)
Private employed	21 (38.9)	33 (61.1)
Commercial sex workers	2 (14.3)	12 (85.7)

### The magnitude of fertility desire and reproductive characteristics

The findings of this study showed that 47.8% of the study subjects have a desire to have children in the future (95% CI, 43%–52.6%). Approximately 74.9% (320) of the women had one to three children; of them, more than two-thirds (66.4%) of the women had no future fertility desire. In total, 54 (12.6%) of the participants did not discuss the issue of fertility desire with healthcare providers despite having a fertility desire to have children in the future (Table 2).

**Table 2:** Magnitude of fertility desire and reproductive characteristics of women living with HIV/AIDS aged 18–49 attending ART units in Arba Minch General Hospital, South Ethiopia, 2021.

Variables		Fertility desire	
		Yes (%)	No (%)
Total number of living children (*N* = 427)	1–2 children	65 (48.1)	70 (51.9)
	≥3 children	68 (36.8)	117 (63.2)
	No live children	71 (66.4)	36 (33.6)
Children's biological sex (*N* = 427)	All boy/s	64 (40.8)	93 (59.2)
	Boy/s and girl/s	43 (48.9)	45 (51.1)
	All girl/s	26 (34.7)	49 (65.3)
	No life children	71 (66.4)	36 (33.6)
Parents pressure to have children (*N* = 427)	Yes	143 (73.7)	51 (26.3)
	No	61 (26.2))	172 (73.8)
Partner's interest to have children (*N* = 427)	Yes	61 (53)	54 (47)
	No	86 (63.7)	49 (36.3)
Community pressure to have children (*N* = 427)	Yes	142 (72.4)	54 (27.6)
	No	62 (26.8)	169 (73.2)
Using contraceptives to prevent pregnancy? (*N* = 427)	Yes	94 (46.3)	109 (53.7)
	No	110 (49.1)	114 (50.9)
History of abortion (*N* = 341)	Yes	25 (44.6)	31 (55.4)
	No	119 (41.8)	166 (58.6)
Discussion with a health professional (*N* = 427)	Yes	54 (47)	61 (53)
	No	150 (48.1)	162 (51.9)

**Figure 1:**
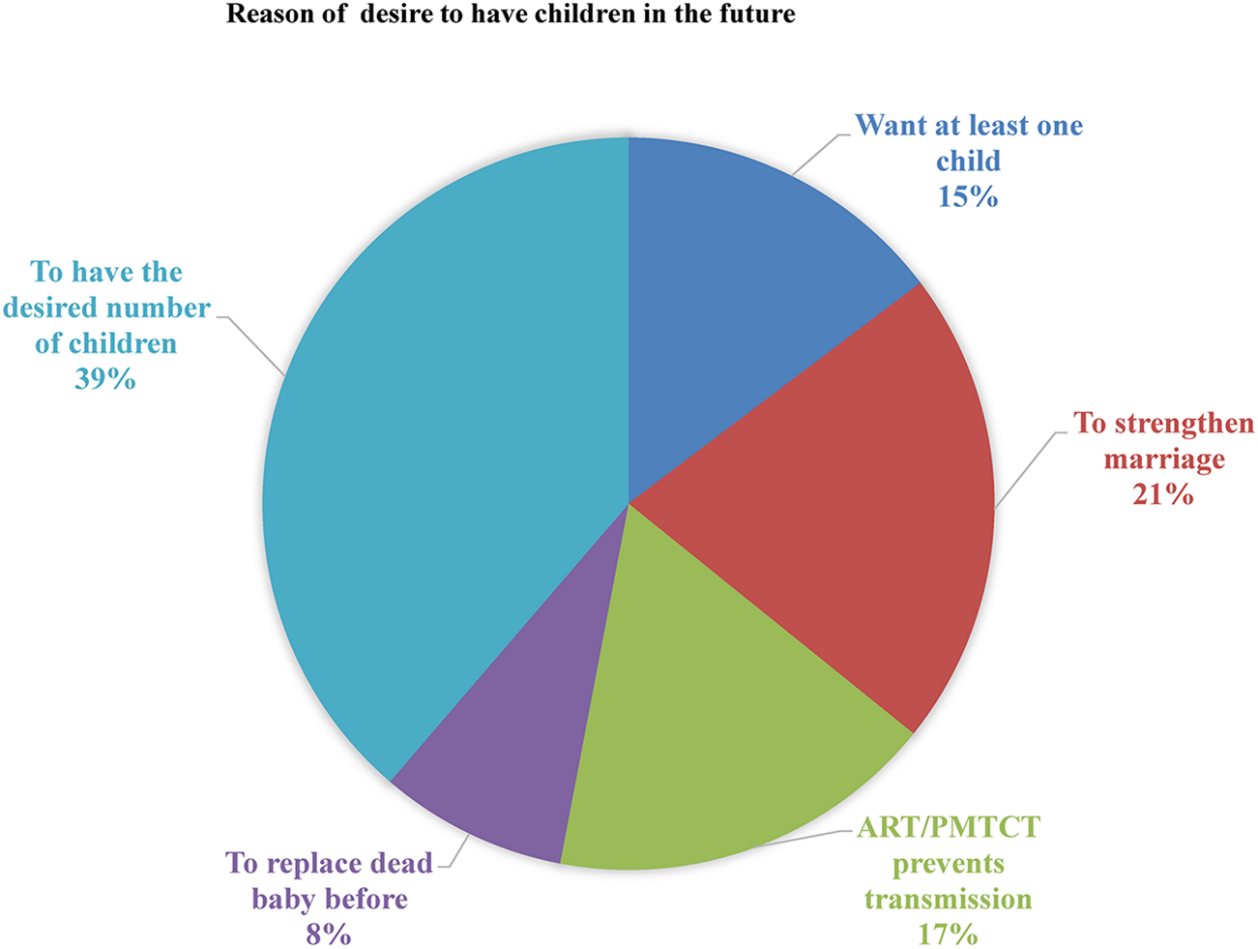
Reason to have the desire to have children in the future among women living with HIV/AIDS attending ART in Arba Minch General Hospital, 2021.

The main reason for their desire to have children was to gain a desired number of children (79, 18.5%), while the main reason *not* to have children was due to the fear of MTCT of HIV (19.4%). In total, 143 (33.5%) of the women who desired to have a child were living with their sexual partner and were sexually active in the last 6 months preceding the study. The majority (285, 66.7%) of those with fertility desires had no history of abortion. Regarding contraceptive utilization, more than one-fourth of the participants (109, 25.5%) had not used contraceptives. The main reason for not using contraceptives was having no husband (137, 32%), while approximately 13.3% (57) of the participants were trying to become pregnant (Figure 1).

### Clinical characteristics

The majority of WLHIV (244, 57.1%) were not voluntarily tested for HIV before knowing their HIV status. More than half of the respondents (225, 52.7%) were on ART for more than 5 years. A total of 314 (73.5%) participants had disclosed their HIV status to anyone, while more than two-thirds (217, 69.1%) of them had not disclosed their HIV status due to fear of stigma (Table 3).

**Table 3:** Clinical characteristics of women living with HIV/AIDS aged 18–49 attending ART units in Arba Minch General Hospital, South Ethiopia, 2021.

Variables		Fertility desire	
		Yes (%)	No (%)
Voluntarily tested for HIV	Yes	93 (50.8)	90 (49.2)
	No	111 (45.5)	133 (54.5)
Initiation of ART (*N* = 427)	<5 years	107 (53)	95 (47)
	≥5 years	97 (43.1)	128 (56.9)
HIV status disclosed to anyone (*N* = 427)	Yes	49 (43.4)	64 (56.6)
	No	155 (49.4)	159 (50.6)
To whom your HIV status disclosed (*N* = 113)	Partner	39 (50.6)	38 (49.40)
	Relative/family	10 (27.8)	26 (72.2)
Reason for not disclosing your HIV status (*N* = 314)	Fear of abuse	18 (69.2)	8 (30.8)
	Fear of divorce	25 (35.2)	46 (64.8)
	Fear of stigma	112 (51.6)	105 (48.4)
Children serostatus (*N* = 427)	Negative	105 (43.4)	138 (56.6)
	Positive	28 (36.4)	49 (63.6)
	No live children	71 (66.4)	36 (33.6)
Do know your recent CD4 count (*N* = 427)	Yes	124 (55.4)	100 (53.6)
	No	80 (39.4)	123 (60.6)
Amount of recent CD4 (*N* = 224)	<200/mm^3^	14 (50)	14 (50)
	≥200/mm^3^	110 (56.1)	86 (43.9)
Do know your viral load (*N* = 427)	Yes	94 (45.4)	113 (54.6)
	No	110 (50)	110 (50)
Amount of recent load (*N* = 208)	<200 copies/ml	11 (45.8)	13 (54.2)
	≥200 copies/ml	83 (45.1)	101 (54.9)
Perceived current health status (*N* = 427)	Improving	162 (48.2)	174 (51.8)
	The same	42 (46.2)	49 (53.8)
Husband's/partner's HIV status (*N* = 250)	Negative	33 (54.1)	28 (45.9)
	Positive	113 (59.8)	76 (40.2)
Husband/partner start ART (*N* = 189)	Yes	115 (6o.2)	75 (39.8)
	No	5 (55.6)	4 (44.4)
Perceived discrimination from a health professional (*N* = 427)	Yes	182 (46.7)	208 (53.3)
	No	22 (59.5)	15 (40.50)
Children lost at any age due to HIV (*N* = 340)	Yes	21 (35.6)	38 (64.4)
	No	122 (43.4)	159 (56.6)

Out of the entire sample, 77 women (18%) were found to have HIV-positive children. Among them, 18 women (36.4%) expressed a desire to have more children in the future. Regarding the participants’ CD4 count, it was found that 193 participants (45.2%) reported a CD4 count exceeding 200/mm^3^, and 184 women (43%) had a viral load that was greater than 200 copies/ml. Of the total participants, 192 individuals (45.1%) desired to have children in the future. The majority of the respondents (336, 78.7%) reported that they perceived an improvement in their health status, and 162 (48.2%) of them desired to have children in the future. A total of 189 participants (47.2%) reported that their husbands/partners were seropositive; among them, 113 individuals (59.8%) desired to have children in the future.

### Prevention of MTCT during pregnancy and lactation

Approximately 364 (85.2%) respondents indicated that HIV transmission to the fetus is not possible during pregnancy. Among this group, 36 respondents (64.3%) had a desire to have children in the future. The majority of the respondents (355, 83.1%) had poor knowledge of HIV prevention and transmission from mothers to their offspring (Table 4).

### Factors associated with fertility desire

In a bivariate analysis, factors such as parental and community pressure to have children, marital status, children’s biological sex, HIV serostatus of children, duration since ART started, perceived discrimination by health professionals, and knowledge of PMTCT were found to be associated with fertility desire (Table 4).

**Table 4:** Knowledge of PMTCT among women living with HIV/AIDS aged 18–49 attending ART units in Arba Minch General Hospital, South Ethiopia, 2021.

Variables		Fertility desire	
		Yes (%)	No (%)
HIV transmit to the fetus during pregnancy	Yes	36 (64.3)	20 (35.7)
	No	166 (45.6)	198 (54.4)
	I don't know	2 (28.6)	5 (71.4)
Can HIV transmit to a child during breastfeeding	Yes	24 (70.6)	10 (29.4)
	No	177 (46)	208 (54)
	I don't know	3 (37.5)	5 (62.5)
Can HIV transmit to a child during labor delivery	Yes	25 (61)	16 (39)
	No	177 (46.8)	201 (53.2)
	I don't know	2 (25)	6 (75)
MTCT during pregnancy	Yes	37 (71.2)	15 (28.8)
	No	160 (44.9)	196 (55.1)
	I don't know	7 (36.8)	12 (63.2)
MTCT during breastfeeding	Yes	37 (66.1)	19 (33.9)
	No	156 (46)	183 (54)
	I don't know	11 (34.4)	21 (65.6)
MTCT during delivery (*N* = 427) What is the infant feeding option of women living with HIV (*N* = 427)	Yes	42 (64.6)	23 (35.4)
	No	148 (46.5)	170 (53.5)
	I don't know	14 (31.8)	30 (68,2)
	Infant formula only	44 (53)	39 (47)
	cow's milk Only	3 (42.9)	4 (57.1)
	Breast milk only for the first sixth month	132 (44.6)	164 (55.4)
	I don't know	25 (61)	16 (39)
Knowledge (*N* = 427)	Poor knowledge	161 (45.4)	194 (55.6)
	Good knowledge	43 (59.7)	29 (40.3)

To control the effects of confounder, a multivariable analysis was conducted. This analysis revealed that factors such as parental pressure to have children, community pressure, being married, having only female children, and having HIV seropositive children were found to be significantly associated with fertility desire.

The odds of fertility desire among WLHIV with parental pressure to have children (AOR: 4.41, 95% CI 2.15–9.05) were 4.41 times higher when compared with those WLHIV without parental pressure to have children. HIV-positive women who had community pressure to have a child (AOR: 2.62, 95% CI 1.30–5.26) were 2.62 times more likely to have fertility desire than women who had no community pressure to have a child. Married HIV-positive women were found to have a 75% less likelihood of fertility desire than HIV-positive women who are not married (AOR = 0.25, 95% CI 0.09–0.73). WLHIV and who had only female children had 2.57 times more likelihood of fertility desire (AOR: 2.577, 95% CI 1.12–5.90) when compared with WLHIV and who had only male children.

HIV-positive women who had HIV seropositive children (AOR: 2.45, 95% CI 1.23–4.85) were 2.45 times more likely to have fertility desire in the future when compared with women who had HIV seronegative children. However, factors such as maternal age, duration since ART started, perceived discrimination by health professionals, knowledge of PMTCT, and education did not have statistically significant association with fertility desire of women (Table 5).

**Table 5:** Bivariate and multivariable analyses of associated factors of fertility desire among WLWHA aged 18–49 attending ART units in Arba Minch General Hospital, South Ethiopia, 2021.

Variables	Had fertility desire		COR (95% CI)	AOR
	Yes (%)	No (%)		
Parent's pressure to have children				
Yes	143 (73.7)	51 (26.3)	7.90 (5.12–12.19)	4.41 (2.15–9.05)^a^
No	61 (26.2)	172 (73.8)	1	1
Community pressure to have children				
Yes	142 (72.4)	54 (27.6)	7.16 (4.67–10.99)	2.62 (1.30–5.26)^a^
No	62 (26.8)	169 (73.2)	1	1
Educational status				
Unable to write and read	12 (36.4)	21 (63.6)	0.38 (0.34–8.22)	0.82 (0.28–2.31)
Read and write	34 (42.5)	46 (57.5)	0.49 (0.44–5.50)	0.62 (0.22–1.70)
Elementary	58 (49.2)	60 (50,8)	0.64 (0.59–4.07)	0.65 (0.22–1.92)
Secondary	38 (44.2)	48 (55.8)	0.52 (0.50–5.10)	0.40 (0.11–1.36)
Preparatory	20 (54.1)	17 (45.9)	0.78 (0.42–3.84)	0.47 (0.15–1.44)
Diploma	30 (56.6)	23 (43.4)	0.87 (0.40–3.27)	0.47 (0.10–2.12)
University level	12 (60)	8 (40)	1	
Marital status				
Single/unmarried	24 (55.8)	19 (44.2)	1	1
Married	149 (59.1)	103 (40.9)	1.14 (0.45–1.67)	0.25 (0.09–0.73)
Widowed	16 (24.2)	50 (75.8)	0.25 (0.24–2.99)	0.82 (0.23–2.84)
Divorced	15 (22.7)	51 (77.3)	0.23 (0.21–4.77)	0.95 (0.43–4.27)
Children's biological sex				
All boy/s	64 (40.8)	93 (59.2)	1	1
Boy/s and girl/s	43 (48.9)	45 (51.1)	1.38 (0.82–2.34)	1.45 (0.74–2.84)
All girl/s	26 (34.7)	49 (65.3)	0.77 (0.65–3.39)	2.57 (1.12–5.90)^a^
No living children	71 (66.4)	36 (33.6)	2.86 (0.27–3.86)	0.26 (0.08–1.79)
HIV serostatus of children				
Negative	105 (43.4)	138 (56.6)	1	1
Positive	28 (36.4)	49 (63.6)	0.75 (0.74–2.26)	2.45 (1.23–4.85)^a^
No living children	71 (66.4)	36 (33.6)	2.59 (0.24–2.62)	0.95 (0.94–5.71)
Duration since ART started				
<5years	107 (53)	95 (47)	1.48 (1.01–2.17)	1.16 (0.70–1.93)
≥5years	97 (43.1)	128 (56.9)	1	1
Perceived discrimination by health professionals				
Yes	182 (46.7)	208 (53.3)	0.59 (0.30–1.18)	0.76 (0.32–1.79)
No	22 (59.5)	15 (40.50)	1	1
Knowledge of PMTCT				
Poor knowledge	161 (45.4)	194 (55.6)	0.56 (0.33–0.93)	0.70 (0.36–1.39)
Good knowledge	43 (59.7)	29 (40.3)	1	1

^a^ *p*-value of <0.05.

## Discussion

The findings of this study showed that 47.8% of the study subjects had the desire to have children in the future. This finding is comparable with the findings of the previous studies conducted in Jima, Tigray, Addis Ababa, and the Oromia region, which reported 46.8%, 45.5%, 44%, and 46.6%, respectively (15–18). In addition, this finding revealed that the fertility desire to have children in the future was similar to the results of a study conducted at the University of Gondar Comprehensive Specialized Hospital with a 52.1% fertility desire (19).

This study showed that fertility desire was higher than what has been reported in most studies previously conducted in other parts of Ethiopia, such as in Finote Selam (33.4%), Amhara region referral Hospitals (40.3%), and South Wollo Zone (18.3%) (10, 20, 21). This discrepancy might be due to service improvements of ARV treatment that restored patient health, and their sexual activity may have increased their fertility desire in this study area since all the studies were conducted 4 years ago. Furthermore, the present study revealed a greater prevalence of fertility desire among WLWHA compared with previous findings in Uganda and the Nairobi Slums, which reported rates of 13.6% and 34%, respectively (22, 23). This difference might be due to improvements in knowledge of PMTCT services in the present study, since the studies in Uganda and Nairobi were conducted 7 and 10 years ago, respectively. During that period, the level of awareness of women toward PMTCT was not as advanced as the findings of the present study.

The findings of this study indicate a smaller percentage of women who had fertility desire (56.2%) compared with the findings of a previous study conducted in the Harari region (11). This discrepancy could potentially be attributed to improvements in the overall health status of HIV-positive women and their diligent adherence to ART in the study area.

This study's findings indicate a decreased prevalence compared with the findings of a previous study conducted in Cameroon (83.3%) and northern Nigeria (65.5%) (24, 25). The findings of this study were observed to be comparatively lower than the findings of previous studies conducted in rural Rakai, Uganda, which reported a fertility desire rate of 63.1% (25). The difference could be attributed to differences in study site, time, socioeconomic status, cultural background, and other health-related factors including adequate information given by healthcare providers regarding the complicated issues surrounding fertility desire to have more children for WLWHA, as well as knowledge of PMTCT. However, this study did not find any statistically significant association in this regard. It may possibly be attributed to the increasing prevalence of HIV/AIDS among the younger reproductive population, who exhibit a strong desire for high fertility.

The findings showed that parents’ pressure to have children was significantly associated with the fertility desire of women living with HIV/AIDS, consistent with the findings of a previous study conducted in Jima (15).

This study found that community pressure to have children was significantly associated with fertility desire, which is similar to that of a study in Jima, Ethiopia (15). This could be due to the fact that in some communities, such as Ethiopia, a child is thought of as a prerequisite for a fulfilled and happy life. In addition, having children is highly valued and is considered a form of social security in the community (20). This finding is also consistent with the findings of previous studies in other countries such as Kenya, where community norms and expectations regarding motherhood influence desires for having more children.

However, most women living with HIV/AIDS perceived that the community discouraged fertility desire among HIV-infected women for three major reasons. First, they feared that an HIV-infected woman might die sooner, and second, they believed that an HIV-infected woman could transmit the infection to her infant. Finally, the worry that childbearing might worsen (26) may have encouraged women living with HIV/AIDS to have more children to demonstrate their health status. However, the health status was not significantly associated in this study.

This study indicated that being married was significantly associated with the fertility desire of women living with HIV/AIDS. Married women demonstrated a 75% less likelihood of expressing desire to have a child compared with those who were not married; this is in agreement with the findings of previous studies conducted in Tigray (27) and consistent with a study conducted in northern Nigeria (28). However, this finding contrasted with the findings of the studies conducted in referral hospitals in the Amhara region, Addis Ababa, Ethiopia, and Osogbo, southwest Nigeria (17, 21, 29, 30). This may be due to the number of children among married women compared with unmarried/single women. The unmarried/single women may desire for children due to cultural influences, as having children is highly valued in the community.

This study revealed that having only female children was significantly associated with the fertility desire of women living with HIV/AIDS, which is in agreement with the findings of previous studies conducted in Jima (15). This might be due to how having sons is considered more valuable than having daughters in the Ethiopian community. The perception of a preference for sons relates to the ascribed ability of sons to support more families by income, provide adequate support to parents in old age, and carry on the family name, while daughters leave their family after marriage.

This study indicated that having seropositive children was a significantly associated factor of fertility desire to have more children among women living with HIV/AIDS, which is an inversely associated factor to the findings of previous studies conducted in Afar (29). This might be due to the fear of losing HIV-infected children in the future from AIDS-related diseases. In addition, it may also stem from the desire of certain women to have more children, with the hope of gaining healthy children. Conversely, women having seronegative children may fear MTCT when having additional children. However, the loss of children after HIV had no significant association with fertility desire in this study. A study conducted in Kenya showed that HIV-positive children were considered as unhealthy, and they have high fertility desire to have HIV-free children in the future if they had HIV seropositive children (25). In contrast, no similar or opposing results were found in existing studies; hence, it is a potential area for further research and a critical area for stakeholders and healthcare providers to increase PMTCT services and discuss fertility desire and its implications with WLWHA.

## Limitation of the study

The study has not included male participants, who are a main patron of fertility desire. Given that the study was conducted in an ART clinic at a health facility, it is possible that individuals who are HIV-positive but not receiving ART were not included in this study.

## Conclusion

Fertility desire in the study area was substantial. Various factors, such as parental pressure, community pressure, only having female children, and the HIV seropositive status of children, contribute to an increase in fertility plans. In contrast, married women had low fertility desire. Policymakers and healthcare providers who are working in an ART clinic should consider the effects of these factors on WLHIV while developing interventions and discussing sexual and reproductive health issues with their clients. Strengthening PMTCT services has the potential to increase the fertility desire of mothers living with HIV/AIDS. By ensuring safe childbirth practices and promoting the avoidance of unintended pregnancies, the likelihood of giving birth to an HIV-free child may motivate WLWHA to have better plans for future pregnancies.

## Data Availability

The original contributions presented in the study are included in the article/**Supplementary Material**, further inquiries can be directed to the corresponding author.
